# FTO Gene Associates and Interacts with Obesity Risk, Physical Activity, Energy Intake, and Time Spent Sitting: Pilot Study in a Nigerian Population

**DOI:** 10.1155/2017/3245270

**Published:** 2017-05-21

**Authors:** Bolaji Fatai Oyeyemi, Charles Ayorinde Ologunde, Ayonposi Bukola Olaoye, Nanfizat Abiket Alamukii

**Affiliations:** ^1^Integrative Biology Unit, Transcriptional Regulation Group, International Centre for Genetic Engineering and Biotechnology, New Delhi, India; ^2^Department of Science Technology, The Federal Polytechnic Ado-Ekiti, Ado-Ekiti, Nigeria; ^3^Institute of Biological, Environmental and Rural Sciences, Aberystwyth University, Aberystwyth, UK; ^4^Cell Biology and Genetics Unit, Department of Zoology, University of Ibadan, Ibadan, Nigeria

## Abstract

Fat mass and obesity-associated (FTO) gene influences obesity but studies have shown that environmental/lifestyle variables like physical activity (PA), time spent sitting (TSS), and energy intake might mediate the effect. However, this is poorly understood in Nigeria due to scarce studies. We demystified association and interaction between FTO rs9939609, obesity, PA, TSS, and energy intake in Nigeria. FTO gene variant was genotyped by restriction fragment length polymorphism and gene sequencing analysis in 103 people with obesity and 98 controls. Anthropometrics and environmental variables were measured using standard procedures. Significant associations were found between FTO rs9939609 with obesity and environmental/lifestyle variables before and after adjusting for age. Carriers of allele A have significantly higher odds of being overweight/obese using BMI [0.191 (0.102–0.361), *p* < 0.001] but this was attenuated by PA (*p*_[interaction]_ = 0.029); odds of being overweight reduced from 0.625 (0.181–2.159) to 0.082 (0.009–0.736) for low and high PA, respectively. Mediation analysis of total indirect effect also confirmed this by showing a simultaneous mediating role of total PA, energy intake, and TSS in the relationship between FTO and BMI (unstandardized-coefficient = 1.68; 95% CI: 1.26–2.22). This study shows a relationship between FTO and obesity phenotype and environmental/lifestyle factors might be an important modulator/mediator in the association.

## 1. Introduction

Increasing number of overweight/obese people has contributed immensely to public health challenge throughout the world. Studies in Nigeria had reported a higher rate of obesity and cardiovascular risk factors [1]. Thus Nigeria is not exempted from the scourge. Therefore, there is the need for greater attention and proactive step to stem the tide. The interactions between environmental and individual factors, including genetic makeup, explain the variability in body size between individuals in a given population [2–5], implying that gene-environment interaction might be germane in the development/progression of overweight/obesity [6].

One of the most known genetic factors predisposing humans to nonmonogenic obesity is a polymorphism in the fat mass and obesity-associated (FTO) gene [7, 8] especially first intron rs9939609 (A/T variant) [9–12]. Individuals carrying this risk allele have been reported to have about 1.09 kg, 0.54 kg/m^2^, and 1.07 cm more weight, BMI, and waist circumference (WC), respectively [10]. However, there are still many unknown attributes regarding the biology of this locus [5, 6].

Human carriers of the susceptible single nucleotide polymorphisms (SNPs) in* FTO *(rs9939609 A/T variant) have also been shown to have a strong relationship with satiety and nutrient preference; this suggests that FTO variant is germane in the dietary macronutrient composition of an individual [5]. To lend credence to this, a recent study suggested that there was FTO (rs9939609 A/T variant) genotype-specific enhancement of neural sensitivity to food stimuli. Thus it seems to be predisposing individual to addiction-supportive food perception [13, 14]. However, Gustavsson et al. [15] reported no evidence of interactions between FTO genotype and macronutrient intake on cardiovascular heart disease risk or BMI. This might be a result of the difference in genetic and environmental variables of their study population; it thus shows that genetic and environmental factors are germane in demystifying obesity.

A high ratio of second to fourth digit finger length (2D : 4D) is widely associated with increased BMI, body size, and coronary heart disease [16–18]. Thus it might be related to the FTO rs9939609 gene variant. Several studies have elucidated the effects of environment and FTO variant on obesity, evident in the well-studied time spent sitting (TSS), physical activity (PA), and dietary correlates of obesity [9, 10, 12, 19]. The first study on FTO gene polymorphism in a well-characterized African (Gambians living in traditional lifestyle) reported that FTO gene variant seems not to influence BMI [20]. But this is slowly being understood in Nigerian context due to limited reports. Studies have shown that some anthropometric variables (ratio of second to the fourth finger (2D : 4D), WC, neck circumference (NC), waist to height ratio (WHtR), and waist to hip ratio (WHR)) might be a surrogate marker for overweight/obesity [21–23]. This study explored those anthropometric traits relationship with FTO rs9939609 variant and the modulating roles of environmental variables (PA, TSS, and energy intake) among young adults. This study combined gene sequencing and restriction fragment length polymorphism (RFLP) to unravel FTO polymorphism in Nigeria population.

## 2. Materials and Methods

This case-control pilot study was conducted among randomly selected unrelated young adults with the mean age of 22.6 (103 people with obesity [BMI ≥ 25.0 Kg/m^2^] and 98 controls) at the Federal Polytechnic Ado-Ekiti (FPA), Nigeria. The study (including informed consent forms and the proposed participant recruitment strategy) was approved by the Research Ethics Committee of the Ekiti State Teaching Hospital Ethical Committee and the FPA Ethical Committee (approval number: FPA/ETH/13-028). We conducted this study in accordance with the Declaration of Helsinki, and all participants gave written informed consent.

Digit length was measured as previously described by Manning et al. [24]. Briefly, subjects kept their hands supine on a flat surface with the palm facing up and the digits straight in the same plane and fingers wide opened in a posture of ease (not kept together tight under artificial pressure).

Body weight, height, WC, HC, and NC were measured with standard procedure while BMI, WHtR, and WHR were calculated as weight (kg) divided by height (m) squared, WC divided by height, and WC divided by HC, respectively.

PA and TSS were measured with self-reported International Physical Activity Questionnaire-Short (IPAQ-SF) form which had been adapted to one of the Nigeria major languages by [25]. Total physical activity was computed and categorized as described by http://www.ipaq.ki.se. Briefly, each category of physical activity was multiplied by their estimated intensity (MET) and reported day(s); the sum of all categories was the total physical activity of the individual. MET intensity includes vigorous (8 METs), moderate (4 METs), and walking (3.3 METs). One MET is the energy dissipated in an inactive state which is 3.5 mL/kg/min of VO_2_ [26].

We used semiquantified food frequency questionnaire (FFQ) to collect information about the dietary habits of our participants as described by [27].

Genomic DNA was isolated from EDTA-anticoagulated whole blood samples using Quick-gDNAMiniPrep kits (ZymoResearch, USA) according to the manufacturer's protocol and stored at –20°C. Genotyping of SNP in rs9939609 was done with PCR-RFLP assay as described by [28]. Briefly, genomic DNA (20 ng) was incubated in a 10 *μ*L solution containing 1 NH4 buffer, 2.5 mmol/liter magnesium, 200 *μ*mol/liter each dNTP, 20 pmol forward (5′-AACTGGCTCTTGAATGAAATAGGATTCAGA-3′) and reverse (5′-AGAGTAACAGAGACTATCCAAGTGCAGTAC-3′) oligonucleotide primers, and 0.5 U Taq DNA polymerase (Inqaba West Africa). The PCR mix was incubated using a touchdown programme at 94°C for 2 minutes followed by 35 cycles of 93°C for 15 seconds, 65°C–56°C for 20 seconds (dropping 0.5°C per cycle), 72°C for 30 seconds, 72°C for 5 minutes, and 10°C *∞*. This was then incubated at 37°C for 3 hours with 2 U ScaI (Inqaba, West Africa). Genotype success rate was 98%.

To confirm results from PCR-RFLP, we proceeded to sequence analysis of the FTO rs9939609 gene: sequencing reactions were performed using the ABI Big Dye Terminator Cycle Sequencing kit version 3.1 (Applied Biosystems Inc., Foster City, CA). Cycling conditions include 94°C/1 minute; 25 cycles of 96°C/10 seconds, and 60°C/4 minutes. Reactions were carried out in a total volume of 20 *μ*L, which included 1–3 ng/*μ*L purified PCR product, 3.2 pmol primer, and 8 *μ*L ABI Prism Big Dye Terminator mix, v3.1 (Applied Biosystems). The sequencing products were cleaned using the ZR-96 DNA Sequencing Clean-up kitTM (Zymo Research Corporation©, Irvine, CA, 2005-2006). Products were analyzed on the Applied Biosystems/Hitachi 3130 9-1-Genetic Analyser. The FTO rs9939609 gene was sequenced bidirectionally in every participant to confirm the observed variants.

### 2.1. Statistical Analysis

Statistical analysis was performed using IBM SPSS 22 and GraphPad Prism 7 for Mac while sample size was computed using G^*∗*^Power (version 3.1; Heinrich Heine University, Düsseldorf, Germany) [29] and method described by Viechtbauer et al. [30] for a pilot study. Power, significance, and minor or risk allele frequencies (MAF) for cases and controls were 95%, 5%, 40%, and 27%, respectively; thus our sample size appears to be sufficient for pilot study.

Data are expressed as frequencies and percentage or as means (standard deviation) unless otherwise stated. Allelic and genotypic frequencies were determined by counting the genotypes and distribution of genotypes in different groups was compared by Chi-square or Fisher exact test. Robust logistic regression was used to evaluate the associations between the FTO rs9939609 genotype (genotype coded as 0 for TT, 1 for AT, and 2 for AA), obesity measures, 2D : 4D, and energy intake (controlled for sex and age). Models were also created for the categorical outcome (overweight/obesity versus control) by robust logistic regression where FTO rs9939609 was included. Analyses were adjusted for sex and age, and odds ratios (OR) with 95% confidence intervals (CI) were calculated to estimate the association between genotypes and measurements. Differences were checked by* T*-test and ANOVA (Significant ANOVA results were further examined using the Bonferroni post hoc test). Differences in data that did not follow a normal distribution (physical activity) were analyzed with nonparametric Kruskal–Wallis and Mann–Whitney tests.

**Figure 1:**
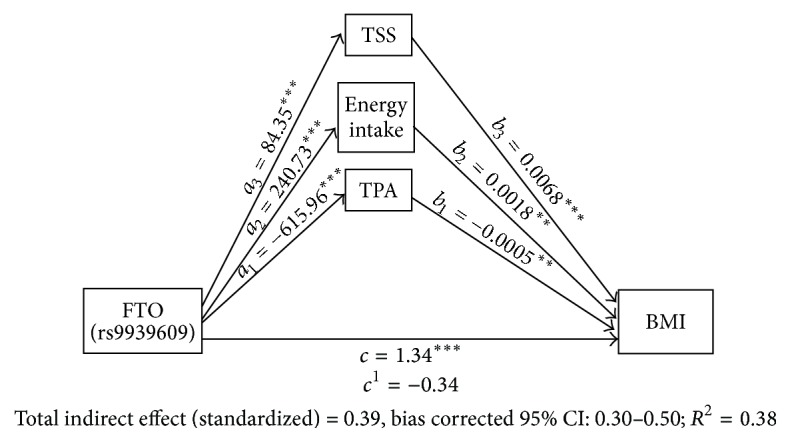
Multiple mediation models of the relationship between FTO rs9939609, TPA, energy intake, TSS, and BMI. (TPA, energy intake, and TSS are mediators). Standardized coefficients are presented and tested for significance with 95% confidence intervals calculated using the bias-corrected bootstrap method (5000 samples).* a* = standardized IV to Med coefficient,* b* = standardized Med to DV coefficient,* c* = standardized total effect (IV to DV), and* c*^1^ = specific indirect effect (indirect path).  ^*∗∗*^*p* < 0.01;  ^*∗∗∗*^*p* < 0.001. BMI: body mass index, TPA: total physical activity, TSS: time spent sitting, IV: independent variable, DV: dependent variable, and Med: mediator.

A statistical method which is known as tests of mediation, which examines the effect of a presumed mediator(s) on the outcome of a predictor on the response variable, is used to investigate possible mediating effects of PA, TSS, and energy intake on the association between our distal variable (FTO rs9939606 genotype) and outcome (BMI). In recent times, studies have employed this method for mediating analysis of mediator(s) on obesity risk allele and BMI [9, 31]. We followed approaches developed by [32] for the multiple mediation analysis on our mediators on the relationship between FTO rs9939609 and BMI. Briefly, we named path coefficient linking the predictor with mediators and path coefficient of a mediator to the outcome as paths *a* and *b*, respectively. Paths* c *and* c*′ refer to the total and indirect effect of FTO rs9939609on BMI in the absence and presence of the mediators, respectively (Figure 1). Bootstrapping was used to create 95% confidence intervals around the “true” value of this cross-product (referred to as the indirect effect). If zero is not within this confidence interval, then the indirect (i.e., mediating) effect is significant. The SPSS “indirect” macro developed to accompany the paper by [32] was used to test the significance. We also checked the mediating effect of each mediator separately and of BMI on the association of FTO rs9939609 on TSS using the same method described above.

A *p* value < 0.05 was considered to have statistical significance.

## 3. Results

We arrived at the sample size with G^*∗*^Power (version 3.1; Heinrich Heine University, Düsseldorf, Germany) [29] and method described by Viechtbauer et al. [30] for a pilot study. Power, significance, and MAF for cases and controls were 95%, 5%, 40%, and 27%, respectively. Thus our sample size appears to be sufficient for this pilot study.

The demographics of the two hundred and one unrelated participants (age range from 17 to 39 years) stratified by sex are presented in Table 1; 51.20%, 32.3%, 53.2%, and 54.7% of individuals were people with obesity using BMI, WC, WHR, and WHtR benchmarks. Cutoff values include BMI ≥ 25.0 kg/m^2^, WC ≥ 80.0 cm for female and ≥94.0 cm for male, and WHR ≥ 0.45 for female and ≥0.5 for male while values ≥0.5 were used for WHtR in both sexes. There was a statistical difference in total physical activity in female and male but not time spent sitting. The ratio of second and fourth digits of both hands was also different (2D : 4D) (Table 1).

All participants were genotyped for FTO SNP rs9939609; thirty-six (17.9%) were homozygous for the obesity risk allele (AA), 90 (44.8%) were heterozygous (AT), and 75 (37.3%) were wild type (TT). The frequency of allele A in this study is 0.40, while it is 0.53 and 0.27 for overweight/obesity and healthy control, respectively. None of these allelic frequencies differs from Hardy-Weinberg equilibrium (*p* > 0.05).

We reported significantly high obesity risk factors in AA, followed by AT and TT, respectively, while the opposite was reported in physical activity pattern (Table 2, Supplementary Table  1, and Supplementary Figures  1–4 in Supplementary material available online at https://doi.org/10.1155/2017/3245270). As shown in Table 3 and Supplementary Figures  1 and 2, presence of at least one copy of the risk allele (A) was associated with some units higher (*β*) obesity risk factors as categorized by body weight (*β*: 3.11 kg per risk allele, 95% CI: 0.39–5.83, *p* < 0.001), BMI (*β*: 1.74 kg/m^2^ per risk allele, 95% CI: 0.89–2.58, *p* < 0.001), WC (*β*: 5.73 cm per risk allele, 95% CI: 3.58–7.88, *p* < 0.001), and NC (*β*: 2.55 cm per risk allele, 95% CI: 1.45–3.65, *p* < 0.001). We also observed that 2D : 4D in both hands showed marked increased in individuals with the risk allele (*β*: 0.018 per risk allele, 95% CI: 0.011–0.025, *p* < 0.001 and *β*: 0.016 per risk allele, 95% CI: 0.010–0.022, *p* < 0.001) for right and left hand, respectively (Table 3 and Supplementary Figure  3). A similar trend was observed for energy intake (354.4 Kcal/day more per risk allele) and TSS (190.5 minutes more per risk allele) (Tables 2 and 3 and Supplementary Figure  4).

**Figure 2:**
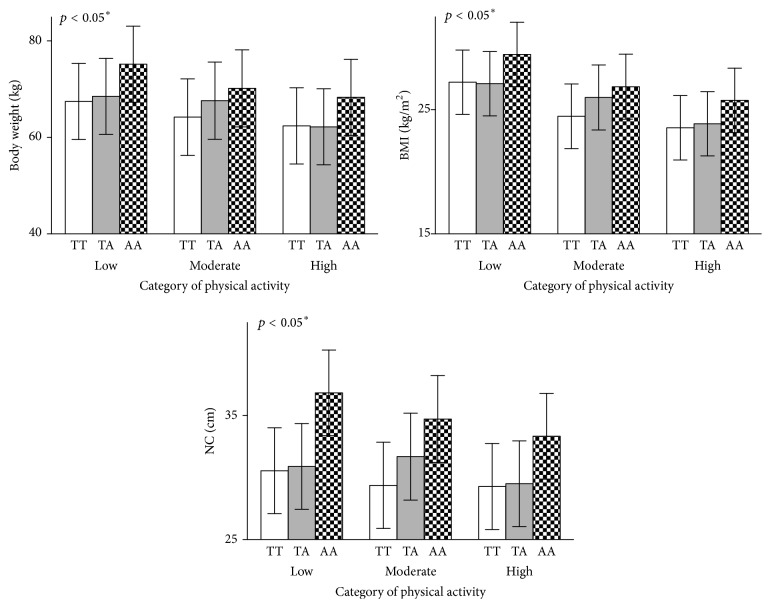
Effect of the FTO rs9939609 genotype on adiposity measures (body weight, BMI, and NC) stratified by category of PA. *p* values are for the interaction between the FTO variant and PA category; least-squares means of different genotypes across all PA groups were calculated using Robust Linear Regression analysis, after adjusting for age and sex. Allele frequency by PA category was low: 20/43/14; moderate: 28/34/13; high: 27/13/9 for TT, TA, and AA genotypes, respectively. BMI: body mass index; NC: neck circumference. ^*∗*^Mean of each variable with genotype for high PA is different from mean of each genotype for low PA at *p* < 0.05 but not in NC TA and TT genotype.

**Figure 3:**
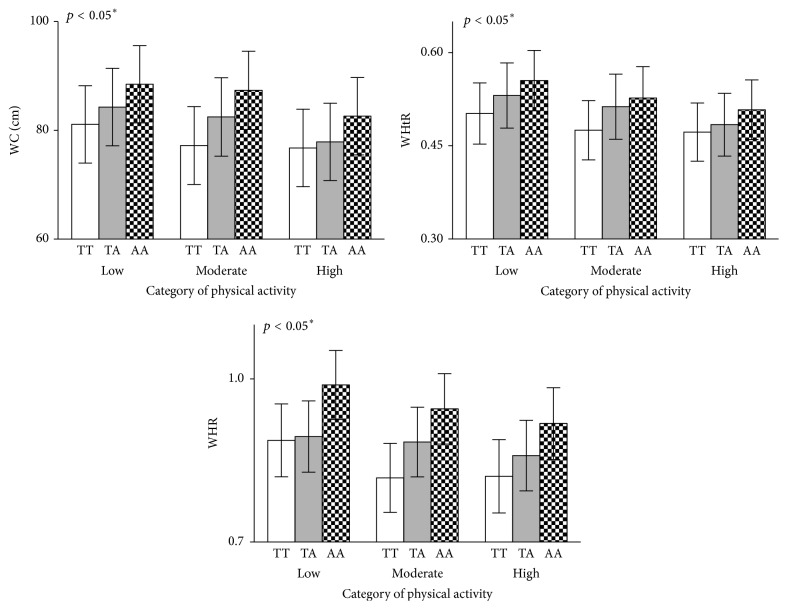
Effect of the FTO rs9939609 genotype on adiposity measures (WC, WHtR, and WHR) stratified by category of PA. *p* values are for the interaction between the FTO variant and PA category; least-squares means of different genotypes across all PA groups were calculated using Robust Linear Regression analysis, with adjustment for age and sex. Allele frequency by PA category was low: 20/43/14; moderate: 28/34/13; high: 27/13/9 for TT, TA, and AA genotypes, respectively. WC: waist circumference, WHtR: waist to height ratio, and WHR; waist to hip ratio. ^*∗*^Mean of each variable with genotype for high PA is different from the mean of each genotype for low PA at *p* < 0.05 but not in WC AA genotype and WHR TA genotype.

**Figure 4:**
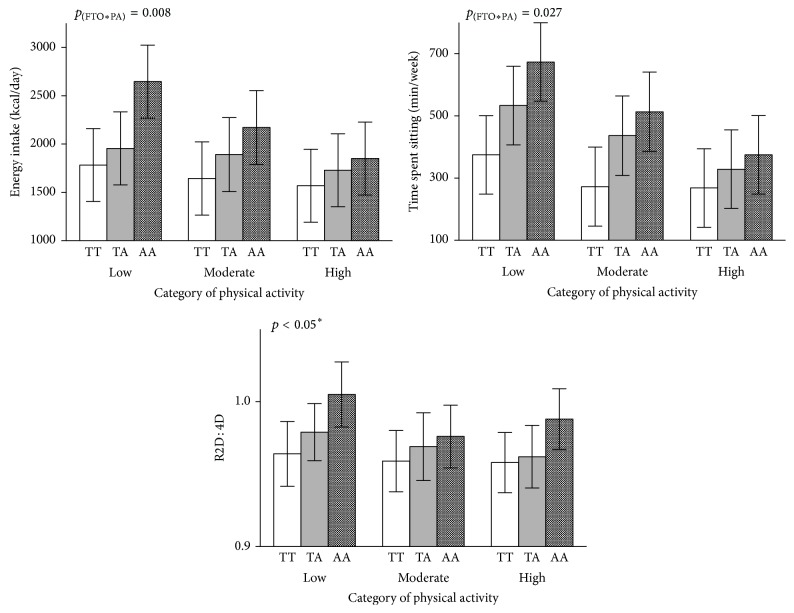
Effect of the FTO rs9939609 genotype on energy intake, TSS, and R2D : 4D stratified by category of PA. *p* values are for the interaction between the FTO variant and PA category; least-squares means of different genotypes across all PA groups were calculated by using Robust Linear Regression analysis, with adjustment for age and sex. Allele frequency by PA category was low: 20/43/14; moderate: 28/34/13; high: 27/13/9 for TT, TA, and AA genotypes, respectively. TSS: time spent sitting. R2D : 4D; ratio of the left hand second digit to the fourth, and TSS; time spent sitting. ^*∗*^Mean of each variable with genotype for high PA is different from mean of each genotype for low PA at *p* < 0.05 but not in R2D : 4D TT and AA genotype.

Participants with FTO risk allele (A) had significantly higher odds of being overweight/obese (OR: 0.19 (0.10–0.36), *p* < 0.001), central obesity (OR: 0.33 (0.15–0.71), *p* = 0.005), and visceral obesity (OR: 0.12 (0.06–0.23), *p* < 0.001) than individuals with the T allele only (Table 3). But the odds were attenuated with increased physical activity levels as shown in Table 4. The odds of overweight/obesity reduced from 0.63 (95% CI: 0.18–2.16) per copy of the risk allele in participants with lower levels of PA to 0.08 (95% CI: 0.009–0.736) in participants with high PA (*p*_(interaction)_ = 0.029) (Table 4). Similar odds trends were found in WHR central obesity (low PA: 0.517 versus high PA: 0.057 per copy of the risk allele, *p*_(interaction)_ = 0.007) and WHtR visceral obesity (low PA: 0.356 versus high PA: 0.142 per copy of the risk allele, *p*_(interaction)_ = 0.010). This was further confirmed in Figures 2–4 where the least square mean of obesity traits in FTO genotypes is different in low and high PA. There was also gene-PA interaction effect on energy intake and time spent sitting with significant *p*_(FTO*∗*PA)_ interaction values of 0.008 and 0.027 was reported, respectively (Figure 4). From the preceding, we might predict that there are mediating effects of PA, TSS, and energy intake on BMI, thus mediation analysis.

Multiple regression analyses were conducted to assess effects of mediator(s) on the relationship between FTO rs9939609 and BMI using mediation model. First, it was found that FTO rs9939609 variant was associated with BMI (*B* = 1.34,* t*(199) = 4.68, and *p* < 0.001) (path *c*). It was also found that FTO rs9939609 variant was significantly related to all mediator TPA (*B* = −615.96,* t*(199) = −5.69, and *p* < 0.001) (path *a*_1_), energy intake (*B* = 240.73,* t*(198) = 5.71, and *p* < 0.001) (path *a*_2_), and TSS (*B* = 84.35,* t*(197) = 5.44, and *p* < 0.001) (path *a*_3_). Lastly, results indicated that all mediator, TPA, energy intake, and TSS were associated with BMI (*B* = −0.0005,* t*(196) = −2.79, and *p* < 0.01 (path *b*_1_); *B* = 0.0018,* t*(196) = 3.99, and *p* < 0.01 (path *b*_2_); and *B* = 0.0068,* t*(198) = 5.33, and *p* < 0.001 (path *b*_3_)), respectively. Because all *a* and *b* paths were significant, multiple mediation analyses were tested using the bootstrapping method with bias-corrected confidence estimates and 95% confidence interval of the indirect effects was obtained with 5000 bootstrap resamples [32].

Results of the mediation analysis of total indirect effect confirmed the simultaneous mediating role of TPA, energy intake, and TSS in the relation between FTO gene variant and BMI (the* total indirect effect *was statistically significant while unstandardized-coefficient = 1.68; 95% CI: 1.26–2.22). Also, results indicated that the direct effect of FTO gene variant on BMI was reduced drastically though significant (*B* = −0.34,* t*(196) = −1.13, and *p* > 0.05) (path *c*^1^) when controlling for mediators, thus suggesting full mediation (Figure 1). Each mediator (TPA, energy intake, and TSS) was tested separately, and they all showed full mediating ability on the interaction between FTO rs9939609 and BMI (Supplementary Figures  5–7). Mediation model was created for the effect of BMI on the association of FTO rs9939609 with TSS and TPA, and it showed that BMI exerts full mediation in both cases (Supplementary Figures  8-9).

## 4. Discussion

In this pilot study, we analyzed SNP rs9939609 of the FTO gene in a group of people with obesity and control in Nigeria. Individuals with the FTO risk allele (A) and low PA level had significantly high obesity risk factors (BMI, WC, NC, WHR, WHtR, body weight, 2D : 4D, energy intake, and TSS). Furthermore, PA, energy intake, and TSS play a mediating role in the effect of the FTO gene variant to BMI. These relationships indicate that the genetic variant and some environmental variables (PA, TSS, and energy intake) might be playing a relevant role in obesity. This study thus elucidated the relationships between FTO genotype, PA, anthropometrics, energy intake, and 2D : 4D in a population residing in Nigeria. But further studies are required to confirm or to refute these observations.

The AA genotype proportion of the FTO rs9939609 reported in this study was significantly higher in people with obesity when compared with control (25.2% to 10.2%); this is in agreement with earlier studies [8, 11, 33]. Our results are in tandem with previous studies as carriers of risk alleles showed higher obesity-related traits [8–11], thus confirming the relationship between FTO rs9939609 and obesity.

This study reported a 1.74 (95% CI 0.47 to 1.10) kg/m^2^ increase in BMI per A allele. This is higher than 1.66, 1.55, 0.79, and 0.54 reported in France, Roma/Gypsy population, and China and across seven European countries [10, 34–36] but lower than 2.8 (95% CI 1.3–6.0) reported in Pakistan female [37]. This shows that our result is comparable to what was reported from other parts of the world. More so, our result is not in agreement with Li et al. [38] that reported no relationship between FTO rs9939609 and BMI in Chinese Han population; this discrepancy might be due to difference in environmental variables, obesity categorization, ethnic difference, and their low MAF, thereby making it having little power to affect obesity in that region. In this study, we reported MAF of 40%, slightly similar to 35.54 reported in Gambia by Hennig et al. [20], higher than (12.1%) reported in China by [36, 38], and lower than (~45%) reported among European population [8].

Similarly, this study shows that FTO rs9939609 A allele carriers had an increased risk (odds) of overweight/obesity [OR = 0.19 (95% CI: 0.10, 0.36); *p* < 0.001], central obesity [OR = 0.33 (95% CI: 0.15, 0.71); *p* < 0.001], and visceral obesity [OR = 0.12 (95% CI: 0.06, 0.23); *p* < 0.001] compared with the TT homozygotes. This is less than the results from other studies across seven European countries [10] and France [34]. The reason might be due to the difference in sample size power, environmental variables, central obesity categorization, and variants in data collection procedure and to different linkage disequilibrium patterns across FTO intron 1 between the various ethnic groups, particularly in populations of African ancestry [4, 5]. Subsequently, our study suggests that the odds of being overweight/obesity was substantially attenuated by active lifestyle (PA). This is similar to other studies that showed that PA might mitigate the effects of gene variant FTO rs9939609 on obesity [10, 19, 36, 39]. But contrary to what was reported in Finland, they reported leisure-time PA [40]. We also reported A allele-dependent increase in energy intake and TSS; this is in line with some recent works [9, 14].

From the foregoing, we might infer that environmental variables (PA, energy intake, and TSS) might have a mediating role in the interaction between FTO rs9939609 and BMI, mediation analysis. Results from multiple mediation analysis showed that all our environmental variables have a full mediating effect on the interaction between FTO rs9939609 and BMI; this is incongruent with other studies by [9, 14, 31].

Studies have shown the importance of both FTO rs9939609 and 2D : 4D as a surrogate marker for obesity and coronary heart disease [8–11, 16–18] but none have studied the relationship between them. We reported an association between FTO gene variant and 2D : 4D with individual carrying FTO risk allele having increased 2D : 4D; thus 2D : 4D might be used to predict individual FTO rs9939609 genotype in future, but further studies are required to buttress and refute this.

Full role of FTO first intron rs9939609 on obesity etiology and their role in energy expenditure need more to be done. Speakman [6] reported that there is inconclusive evidence (like epistatic or gene-environment interaction) in the relationship between FTO, PA, and energy expenditure. Thus, we can explore gene-environment interaction to demystify biology of FTO.

This study presents evidence about the role of genetic variant and gene-environment interaction concerning obesity in a Nigerian youth population which is based case-control study with good response rate and low selection bias. Also, our study had the power to detect the association of rs9939609 with obesity in Nigerian youth. However, several limitations should also be noted. First, this study was carried out in a Polytechnic campus which might affect response to some parameters like PA because the layout of the school seems to encourage PA. Second, we need an objective measure of environmental variables like accelerometer for PA to be sure of the PA level. Lastly, only one SNP of* FTO* was measured in this study, so it is unclear whether the identified association is due to this particular sequence variant or another variant in tight linkage disequilibrium with rs9939609 [40].

Power value (95%) and Bonferroni correction for multiple testing used in this study reduce both the risk of false positive and false negative results; thus we might draw some conclusion from our results. Nigeria relies on results from other parts of the world on the interaction of genetic and environmental impact on obesity; this might not be good because studies have shown variations from the different ethnic population [2–5]. Thus this study made an indigenous genetic study of obesity available, thus paving the way for proper management of obesity because PA, TSS, and energy intake showed modulating/mediating effect on the interaction between FTO and obesity. Lastly, such evidence of gene-environmental/lifestyle interaction will be of great importance because environment/lifestyle can be modified easily unlike gene that is inherent in the particular population.

## 5. Conclusions

Our study suggests that environmental/lifestyle factors like PA, TSS, and energy intake might be substantial modulator and/or mediator in the association between FTO rs9939609 and BMI in Nigeria because they mediate the influence of FTO variant on BMI, offering new insight into the interrelationship between FTO genetic variants, TPA, energy intake, and TSS in Nigeria. Our data attest the previously reported association regarding the genetic variability in intron 1 of FTO gene and obesity risk. This might propose a mechanism of understanding of FTO-linked weight gain and obesity in the Nigerian context, but further study with larger sample size and objective measures of environmental variable is warranted.

## Supplementary Material

Supplementary Figure 1: Association between FTO rs9939609 genotype and adiposity measures (body weight, BMI and NC). Supplementary Figure 2: Association between FTO rs9939609 genotype and adiposity measures (WC, WHtR and WHR). Supplementary Figure 3: Association between FTO rs9939609 genotype and adiposity measures (R2D : 4D and L2D : 4D). Supplementary Figure 4: Association between FTO rs9939609 genotype, Energy intake and TSS. Supplementary Figure 5: Mediation effect of TPA on the association of FTO (rs9939609) with BMI using mediation model. Supplementary Figure 6: Mediation effect of energy intake on the association of FTO (rs9939609) with BMI using mediation model. Supplementary Figure 7: Mediation effect of TSS on the association of FTO (rs9939609) with BMI using mediation model. Supplementary Figure 8: Mediation effect of BMI on the association of FTO (rs9939609) with TPA using mediation model. Supplementary Figure 9: Mediation effect of BMI on the association of FTO (rs9939609) with TSS using mediation model. Supplementary Table 1: Anthropometric traits of overweight and control subjects stratified by FTO rs6639609 variants.

## Figures and Tables

**Table 1:** Characteristics of the study population.

Characteristics	Overall (*n* = 201)	Female (*n* = 102)	Male (*n* = 99)	*p*
Age (years)	22.64 (3.61)	23.05 (3.69)	22.22 (3.50)	0.105
Anthropometric				
Body weight (kg)	66.98 (9.54)	62.54 (7.80)	71.56 (9.03)	<0.001
BMI (kg/m^2^)	25.96 (3.06)	25.52 (3.15)	26.41 (2.91)	0.044
Normal (*n*; %)^a^	98; 48.80	45; 22.40	53; 26.40	0.182
Overweight/obesity (*n*; %)^b^	103; 51.20	57; 28.40	46; 22.90	0.322
NC (cm)	31.24 (4.00)	30.78 (5.06)	31.25 (4.34)	0.121
WC (cm)	81.67 (8.02)	80.91 (7.50)	82.47 (8.48)	0.169
Central obesity (*n*; %)^c^	65; 32.30	56; 27.90	9; 4.50	<0.001
WHR	0.881 (0.081)	0.862 (0.070)	0.899 (0.088)	0.001
Central obesity (*n*; %)^d^	107; 53.20	57; 28.40	50; 24.90	0.445
WHtR	0.507 (0.055)	0.514 (0.053)	0.499 (0.056)	0.038
Visceral obesity (*n*; %)^e^	110; 54.70	62; 30.80	48; 23.90	0.08
R2D : 4D	0.9711 (0.027)	0.9819 (0.027)	0.9600 (0.022)	<0.001
L2D : 4D	0.9684 (0.024)	0.9791 (0.023)	0.9573 (0.021)	<0.001
Energy intake (kcal/day)^f^	1848.76 (431.17)	1763.73 (397.96)	1936.36 (448.23)	0.004
Time spent siting (minutes/week)	411.19 (183.12)	400.98 (173.53)	421.72 (192.82)	0.424
Physical activity (PA)				
Total PA (MET-minutes/week)^*∗∗*^	678 (4177–40)	643.5 (3405–40)	1335 (4177–188.5)	<0.001
Light PA (MET-minutes/week)^*∗∗*^	396 (1782–0)	280.5 (1782–0)	495 (1782–33)	<0.001
Moderate PA (MET-minutes/week)^*∗∗*^	180 (1800–0)	120 (1800–0)	320 (1680–0)	<0.001
Vigorous PA (MET-minutes/week)^*∗∗*^	80 (1800–0)	0 (1800–0)	80 (1800–0)	0.072

Data are presented as means (standard deviation (SD) values shown in parenthesis) except stated otherwise. The difference was evaluated using Student's *T*-test and Chi-square test for continuous and categorical variables, respectively; BMI: body mass index, NC: neck circumference, WC: waist circumference, WHR: waist to hip ratio, WHtR: waist to height ratio, R2D : 4D: ratio of right hand second to fourth digit, and L2D : 4D: ratio of left hand second to the fourth digit; *n* = number of samples; % = percentage; ^a^normal BMI < 25 kg/m^2^ while ^b^overweight/obesity BMI ≥ 25 kg/m^2^; ^c^central obesity was defined as WC > 80 cm for female and > 94 cm for male; ^d^central obesity was defined as WHR > 0.85 for female and >0.90 for male; ^e^visceral obesity was defined as WHtR > 0.5; ^f^total energy intake per day from carbohydrate sources; ^*∗∗*^data expressed by median (range); Mann–Whitney *U* test.

**Table 2:** Anthropometric traits of overweight and control subjects stratified by FTO rs6639609 variants.

Characteristics	Overall (*n* = 201)	TA (*n* = 90)	AA (*n* = 36)	*p*
	TT (*n* = 75)			
Body weight (kg)	65.03 (8.04)^a^	66.67 (10.49)^a^	71.82 (8.47)	0.007^a^
BMI (kg/m^2^)	24.95 (2.50)^a^	26.10 (3.24)^a^	27.70 (2.88)	0.006^a^
NC (cm)	29.72 (2.63)^a^	30.91 (3.78)^a^	35.20 (4.39)	<0.001^a^
WC (cm)	78.20 (7.14)	82.70 (7.46)	86.33 (8.21)	0.026
WHtR	0.481 (0.05)	0.519 (0.05)^a^	0.532 (0.06)^a^	0.006^a^
WHR	0.840 (0.07)	0.883 (0.07)	0.957 (0.08)	0.002
R2D : 4D	0.9586 (0.02)	0.9742 (0.02)	0.9894 (0.04)	0.0026
L2D : 4D	0.9570 (0.02)	0.9726 (0.02)	0.9816 (0.03)	0.013
Energy intake (kcal/day)	1662.00 (377.76)	1891.67 (370.25)	2273.89 (537.91)	0.008
TSS (minutes/week)	300.13 (129.75)	465.22 (151.73)	541.94 (160.62)	0.003
TPA^*∗∗*^	1668.00 (4177–99)	625.25 (4120–40)	645.00 (2826–89.5)	<0.001
VPA^*∗∗*^	792.00 (1782–0)	297.00 (1782–0)	231.00 (1386–33)	<0.001
MPA^*∗∗*^	480.00 (1800–0)	170.00 (1760–0)	120.00 (1440–0)	<0.001
LPA^*∗∗*^	96.00 (1800–0)	0 (1680–0)	0 (1800–0)	0.066

Data are expressed as mean (SD values presented in parenthesis) except stated otherwise; ANOVA test (Bonferroni multiple comparability test) was used to compare means across genotype while Kruskal–Wallis test was used to test the difference in physical activity; BMI: body mass index, NC: neck circumference, WC: waist circumference, WHR: waist to hip ratio, WHtR: waist to height ratio, R2D : 4D: ratio of right hand second to fourth digit, and L2D : 4D: ratio of left hand second to fourth digit; TSS: time spent sitting, TPA: total physical activity, VPA: vigorous physical activity, MPA: moderate physical activity, and LPA: low physical activity; ^a^data expressed with the same superscript in the same row are not statistically different at *p* < 0.05; ^*∗∗*^data are expressed by median (range); Kruskal–Wallis test (comparability test Dunns).

**Table 3:** Association of FTO rs9939609 genotype with obesity-related traits.

Variables	Estimated change per unit A allele (95% CI)	*p*
Anthropometrics		
Body weight (kg)	3.109 (0.392–5.827)	0.025
BMI (kg/m^2^)	1.735 (0.89–2.579)	<0.001
WC (cm)	5.73 (3.58–7.88)	<0.001
WHR	0.068 (0.047–0.089)	<0.001
WHtR	0.041 (0.026–0.055)	<0.001
NC (cm)	2.55 (1.45–3.65)	<0.001
Overweight or obesity^a^	0.191 (0.102–0.361)	<0.001
Central obesity^b^	0.328 (0.151–0.714)	0.005
Central obesity^c^	0.131 (0.068–0.253)	<0.001
Visceral obesity^d^	0.12 (0.062–0.232)	<0.001
Energy intake (kcal/day)	354.40 (231.71–477.10)	<0.001
TSS (minutes/week)	190.52 (147.8–233.23)	<0.001
R2D : 4D	0.018 (0.011–0.025)	<0.001
L2D : 4D	0.016 (0.01–0.022)	<0.001

Data presented as beta coefficient (for continuous outcome) or odd ratio (OR) (for binary outcome) with corresponding 95% confidence interval (CI). Models were adjusted for age and sex; BMI: body mass index, NC: neck circumference, WC: waist circumference, WHR: waist to hip ratio, WHtR: waist to height ratio, R2D : 4D: ratio of the right hand second digit to the fourth, and L2D : 4D: ratio of the left hand second digit to the fourth; ^a^overweight or obesity was defined as BMI ≥ 25.0 kg/m^2^; ^b^central obesity was defined as WC > 80 cm for female and >94 cm for male; ^c^central obesity was defined as WHR > 0.85 for female and >0.90 for male; ^d^visceral obesity was defined as WHtR > 0.5.

**Table 4:** Association between FTO rs9939609 genotype and obesity measures by category of PA.

Variable	Low PA (*n* = 77)	*p*	Moderate PA (*n* = 75)	*p*	High PA (*n* = 49)	*p*	*p*_(FTO*∗*PA)_
	OR (95% CI)		OR (95% CI)		OR (95% CI)		
Overweight^a^	0.625 (0.181–2.159)	0.46	0.113 (0.033–0.387)	0.001	0.082 (0.009–0.736)	0.026	0.029
Central obesity^b^	0.78 (0.212–2.872)	0.71	0.273 (0.065–1.151)	0.077	0.187 (0.037–0.952)	0.043	0.345
Central obesity^c^	0.517 (0.174–1.539)	0.24	0.047 (0.011–0.194)	<0.001	0.057 (0.01–0.323)	0.001	0.007
Visceral obesity^d^	0.356 (0.116–1.089)	0.07	0.039 (0.01–0.16)	<0.001	0.142 (0.032–0.636)	0.011	0.010

Data presented as the odd ratio (OR) with corresponding 95% confidence interval (CI). Models were adjusted for age and sex; BMI: body mass index, WC: waist circumference, WHR: waist to hip ratio, and WHtR: waist to height ratio; ^a^overweight or obesity was defined as BMI ≥ 25.0 kg/m^2^;  ^b^central obesity was defined as WC > 80 cm for female and >94 cm for male; ^c^central obesity was defined as WHR > 0.85 for female and >0.90 for male; ^d^visceral obesity was defined as WHtR > 0.5.
