# Supplementary material for: FTO Gene Associates and Interacts with Obesity Risk, Physical Activity, Energy Intake, and Time Spent Sitting: Pilot Study in a Nigerian Population
Source: J Obes. 2017 May 21;2017:3245270. doi: 10.1155/2017/3245270 (PMC5457775; doi:10.1155/2017/3245270)
Supplement: Supplementary file 1 — Supplementary Figure 1: Association between FTO rs9939609 genotype and adiposity measures (body weight, BMI and NC). Supplementary Figure 2: Association between FTO rs9939609 genotype and adiposity measures (WC, WHtR and WHR). Supplementary Figure 3: Association between FTO rs9939609 genotype and adiposity measures (R2D : 4D and L2D : 4D). Supplementary Figure 4: Association between FTO rs9939609 genotype, Energy intake and TSS. Supplementary Figure 5: Mediation effect of TPA on the association of FTO (rs9939609) with BMI using mediation model. Supplementary Figure 6: Mediation effect of energy intake on the association of FTO (rs9939609) with BMI using mediation model. Supplementary Figure 7: Mediation effect of TSS on the association of FTO (rs9939609) with BMI using mediation model. Supplementary Figure 8: Mediation effect of BMI on the association of FTO (rs9939609) with TPA using mediation model. Supplementary Figure 9: Mediation effect of BMI on the association of FTO (rs9939609) with TSS using mediation model. Supplementary Table 1: Anthropometric traits of overweight and control subjects stratified by FTO rs6639609 variants. [file 3245270.f1.pdf]

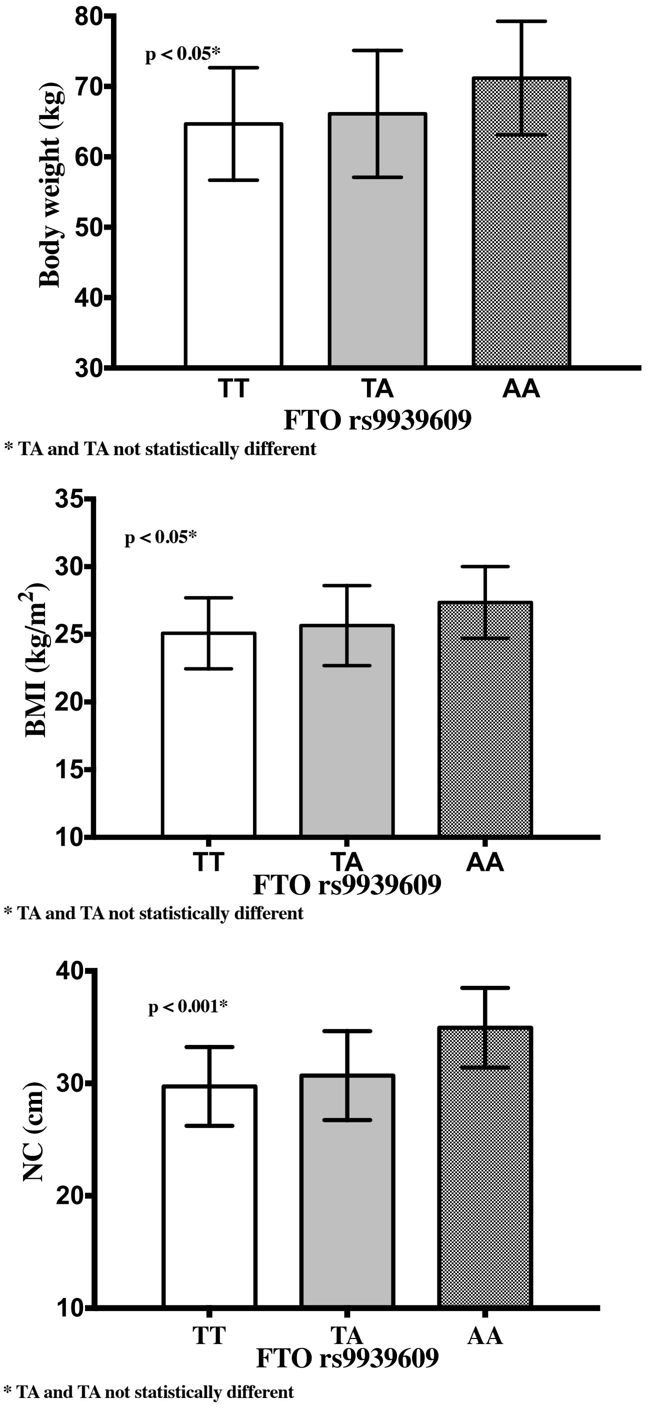

Supplementary Figure 1. Association between FTO rs9939609 genotype and adiposity measures (body weight, BMI and NC). Least-squares means of genotypes were calculated by using Robust Linear Regression, with adjustment for age and sex.

BMI: Body mass index, NC: Neck circumference

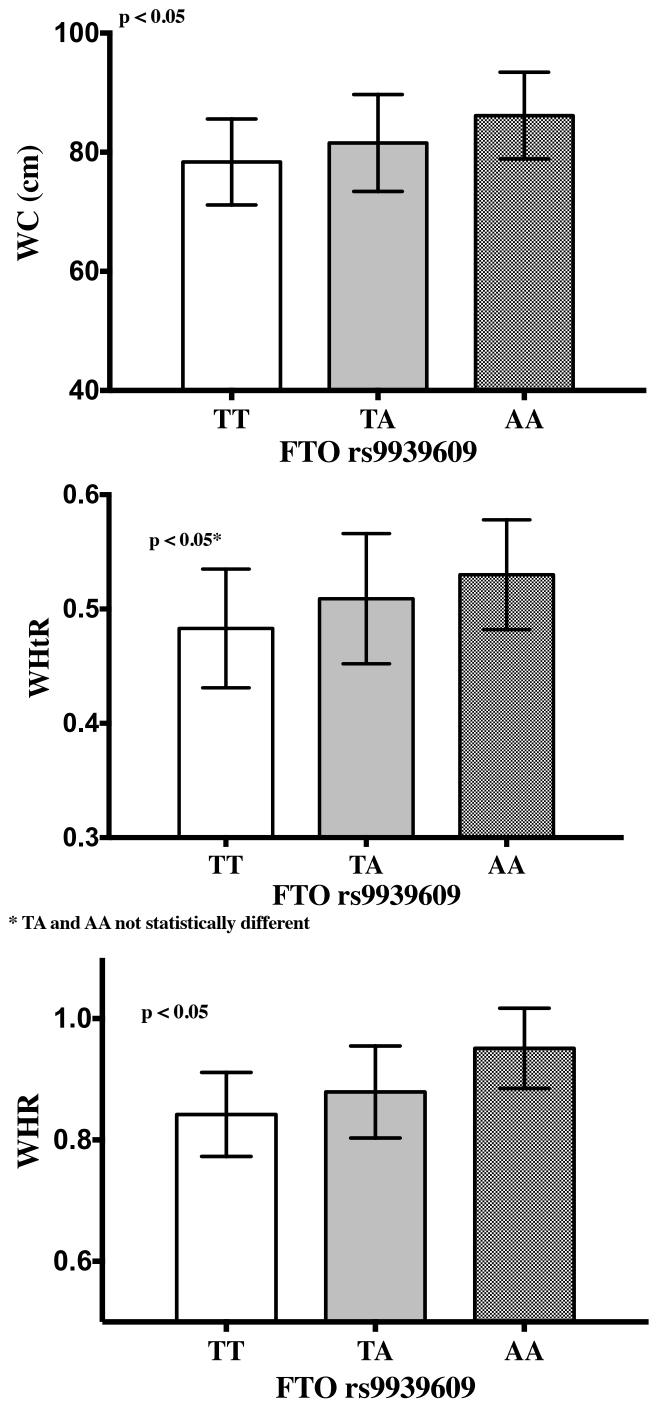

Supplementary Figure 2. Association between FTO rs9939609 genotype and adiposity measures (WC, WHtR and WHR). Least-squares means of genotypes were calculated by using Robust Linear Regression, with adjustment for age and sex.

WC: Waist circumference, WHtR: Waist to height ratio, WHR; Waist to hip ratio

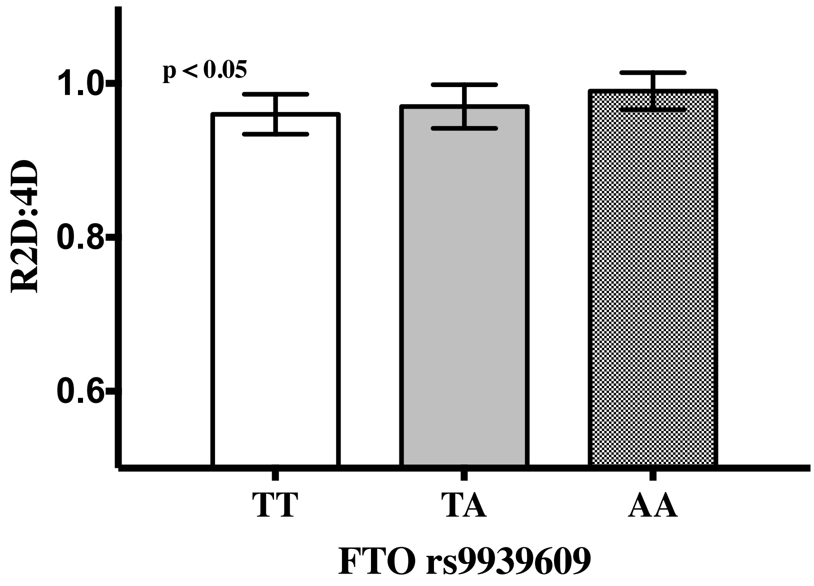

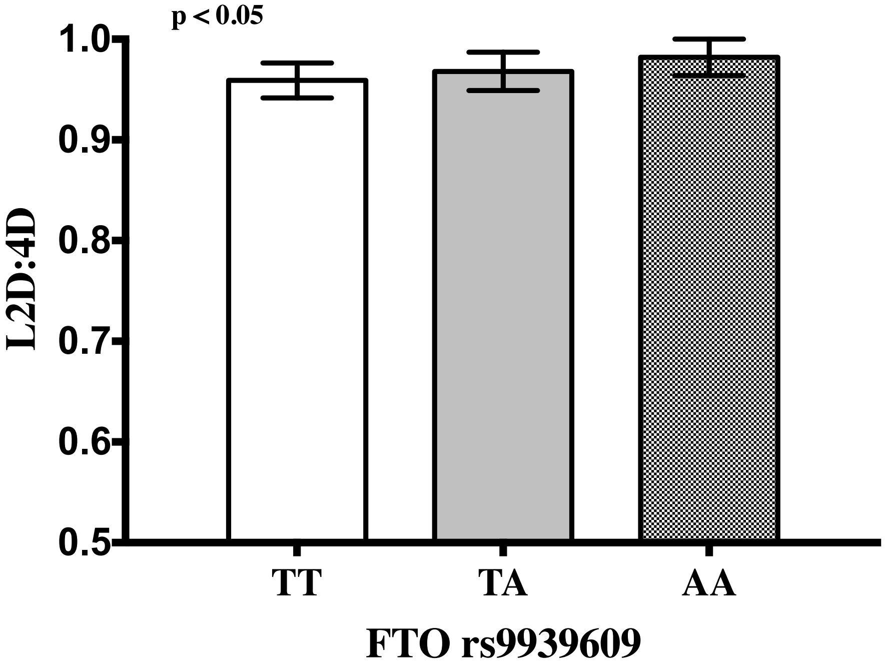

Supplementary Figure 3. Association between FTO rs9939609 genotype and adiposity measures (R2D:4D and L2D:4D). Least-squares means of genotypes were calculated by using Robust Linear Regression, with adjustment for age and sex.

R2D:4D and L2D:4D: Ratio of second to forth digit for right and left hand respectively

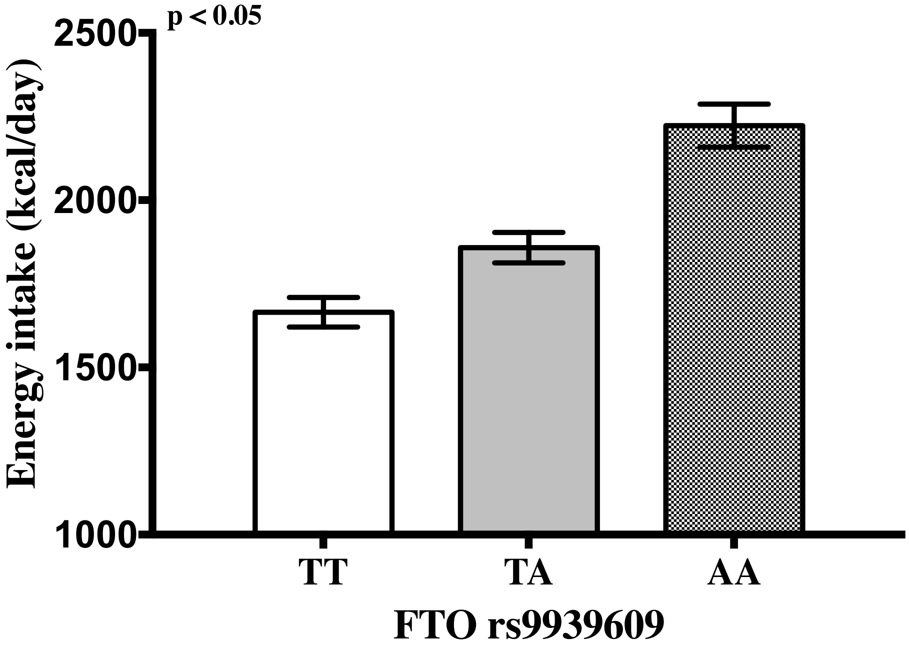

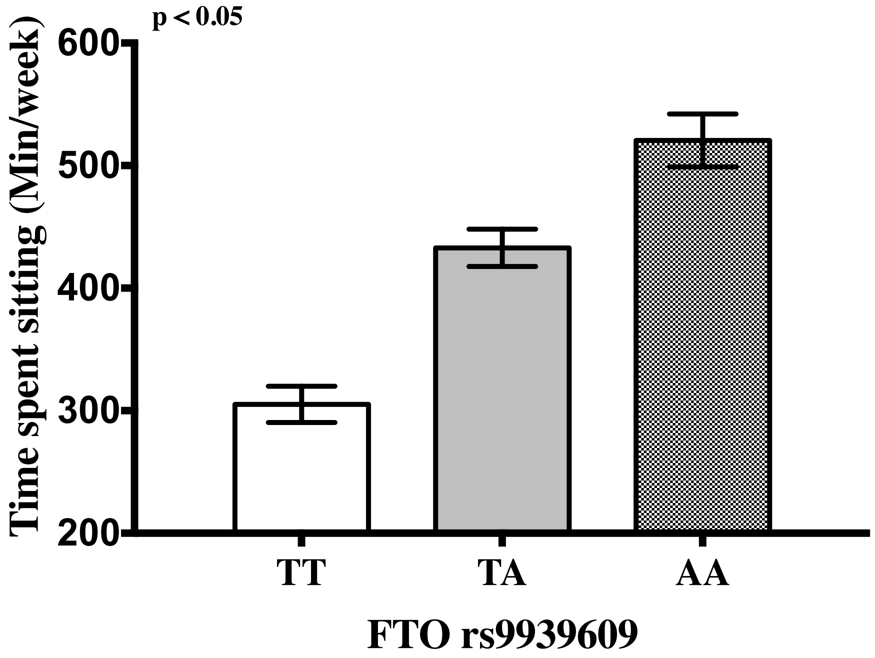

Supplementary Figure 4. Association between FTO rs9939609 genotype, Energy intake and TSS. Least-squares means of genotypes were calculated by using Robust Linear Regression, with adjustment for age and sex.

TSS: Time spent sitting

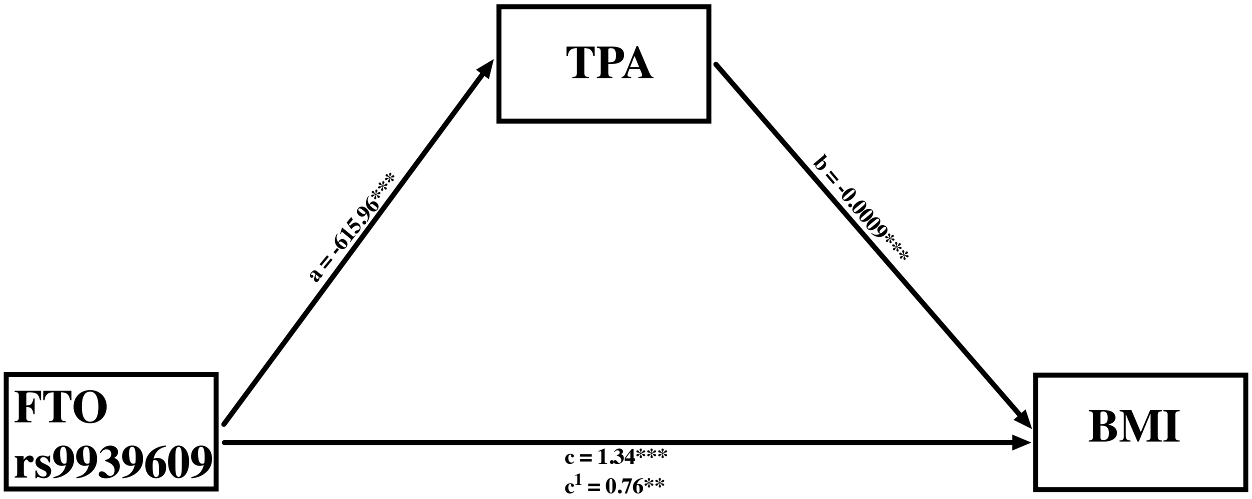

Total indirect effect (Standardized) = 0.13, bias corrected 95% CI: 0.08-0.199; R^2 = 0.214

Supplementary Figure 5. Mediation effect of TPA on the association of FTO (rs9939609) with BMI using mediation model.

Standardized coefficients are presented and tested for significance with 95% confidence intervals calculated using the bias-corrected bootstrap method (5000 samples). a = standardized IV to Med coefficient, b = standardized Med to DV coefficient, c = standardized total effect (IV to DV), c^1 = specific indirect effect (indirect path).

* $p < 0.05$ ** $p < 0.01$ *** $p < 0.001$

BMI: Body mass index, TPA: Total physical activity, IV: Independent variable, DV: Dependent variable and Med: Mediator

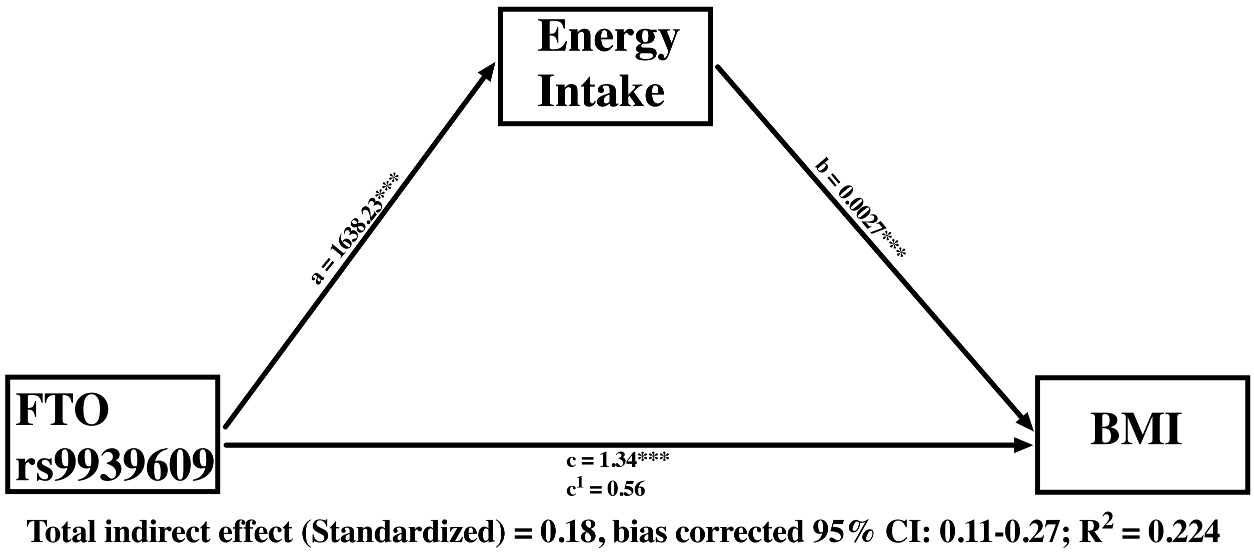

Supplementary Figure 6. Mediation effect of energy intake on the association of FTO (rs9939609) with BMI using mediation model.

Standardized coefficients are presented and tested for significance with 95% confidence intervals calculated using the bias-corrected bootstrap method (5000 samples). a = standardized IV to Med coefficient, b = standardized Med to DV coefficient, c = standardized total effect (IV to DV), c^1 = specific indirect effect (indirect path).

* $p < 0.05$ ** $p < 0.01$ *** $p < 0.001$

BMI: Body mass index, IV: Independent variable, DV: Dependent variable and Med: Mediator

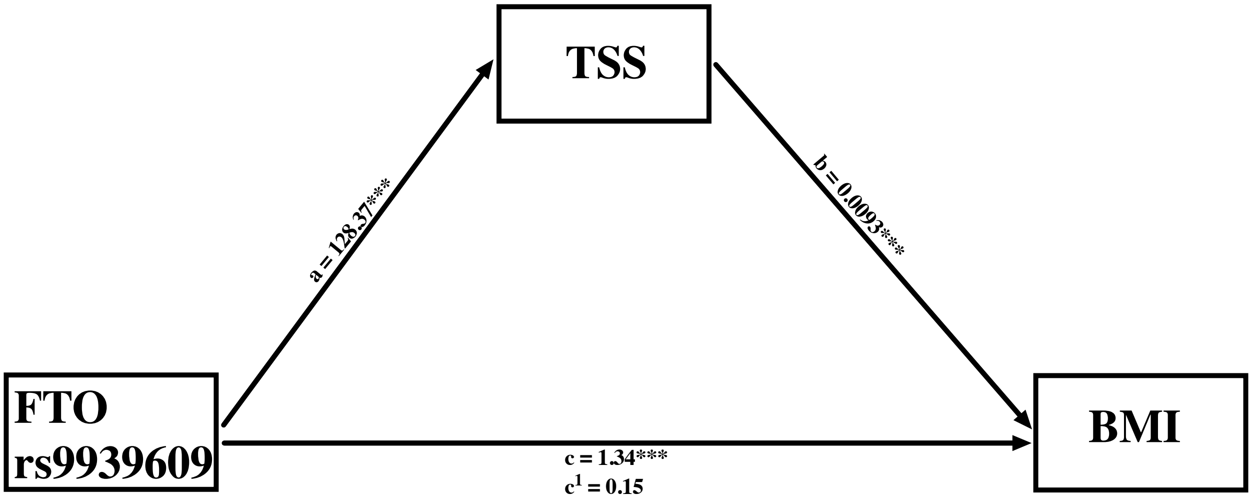

Total indirect effect (Standardized) = 0.28, bias corrected 95% CI: 0.20-0.38; R^2 = 0.30

Supplementary Figure 7. Mediation effect of TSS on the association of FTO (rs9939609) with BMI using mediation model.

Standardized coefficients are presented and tested for significance with 95% confidence intervals calculated using the bias-corrected bootstrap method (5000 samples). a = standardized IV to Med coefficient, b = standardized Med to DV coefficient, c = standardized total effect (IV to DV), c^1 = specific indirect effect (indirect path).

* $p < 0.05$ ** $p < 0.01$ *** $p < 0.001$

BMI: Body mass index, TSS: Time spent sitting, IV: Independent variable, DV: Dependent variable and Med: Mediator

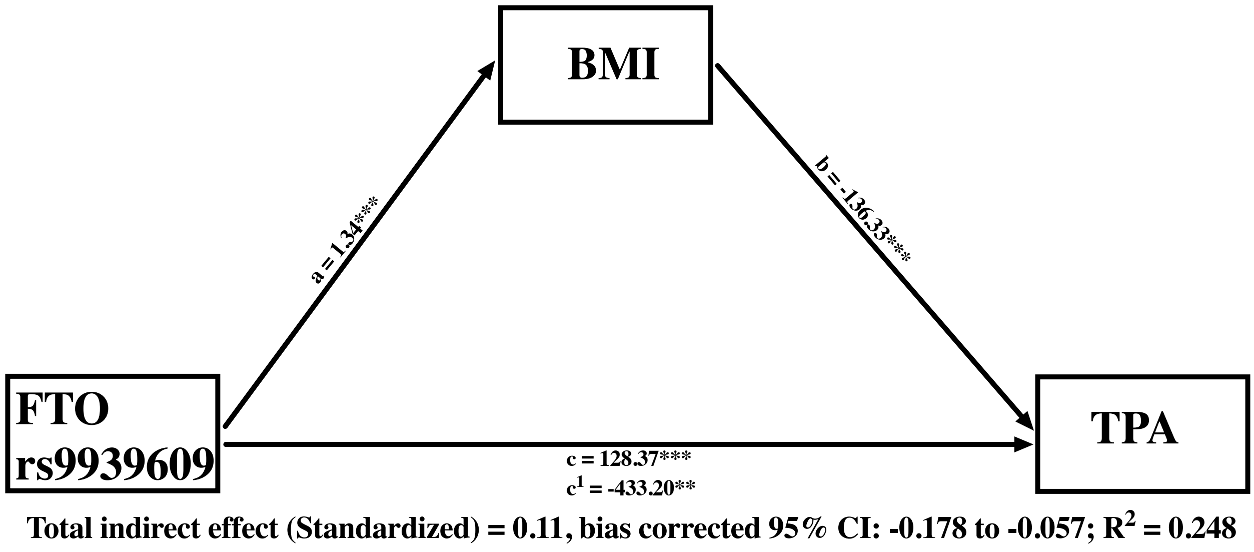

Supplementary Figure 8. Mediation effect of BMI on the association of FTO (rs9939609) with TPA using mediation model.

Standardized coefficients are presented and tested for significance with 95% confidence intervals calculated using the bias-corrected bootstrap method (5000 samples). a = standardized IV to Med coefficient, b = standardized Med to DV coefficient, c = standardized total effect (IV to DV), c^1 = specific indirect effect (indirect path).

* $p < 0.05$ ** $p < 0.01$ *** $p < 0.001$

BMI: Body mass index, TPA: Total physical activity, IV: Independent variable, DV: Dependent variable and Med: Mediator.

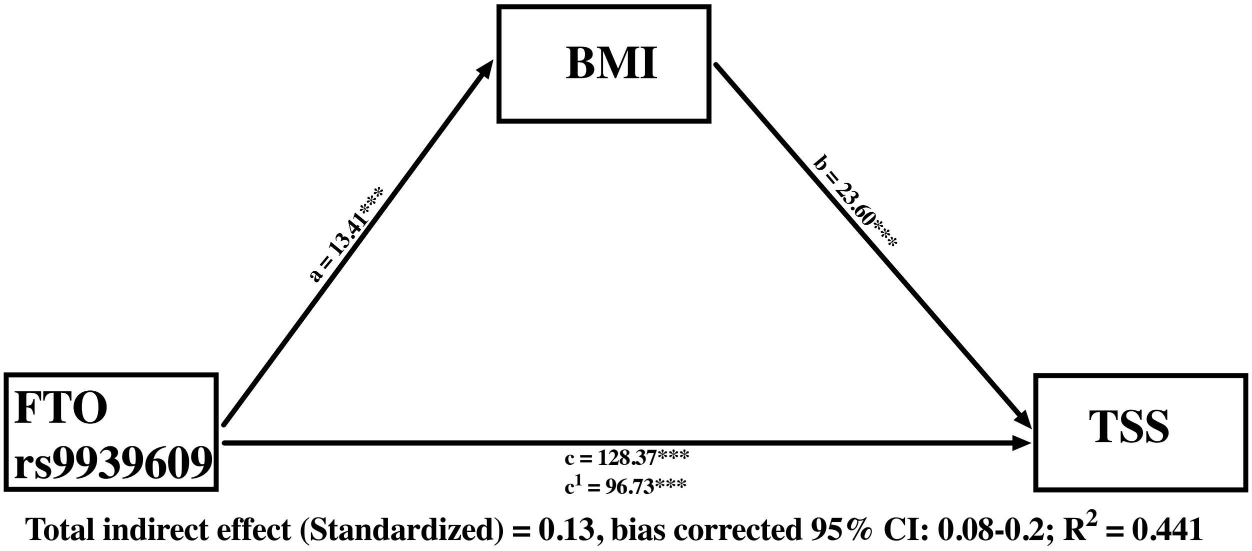

Supplementary Figure 9. Mediation effect of BMI on the association of FTO (rs9939609) with TSS using mediation model.

Standardized coefficients are presented and tested for significance with 95% confidence intervals calculated using the bias-corrected bootstrap method (5000 samples). a = standardized IV to Med coefficient, b = standardized Med to DV coefficient, c = standardized total effect (IV to DV), c^1 = specific indirect effect (indirect path).

* $p < 0.05$ ** $p < 0.01$ *** $p < 0.001$

BMI: Body mass index, TSS: Time spent sitting, IV: Independent variable, DV: Dependent variable and Med: Mediator.

Supplementary Table 1: Anthropometric traits of overweight and control subjects stratified by FTO rs6639609 variants

	Control (n = 98)				Overweight/obesity (n = 103)			
	TT (n=55)	TA(n=33)	AA(n=10)	p	TT(n=20)	TA(n=57)	AA(n=26)	p
Body weight (kg)	63.59(7.61)[a]	58.36(8.32)	67.34(5.83)[a]	0.011*	69.00(8.05)	71.48(8.43)	73.54(8.77)	0.49
BMI (kg/m^2)	23.65(0.97)	22.56(1.77)	23.73(1.06)	0.414	28.50(1.80)	28.15(1.81)	29.23(1.58)	0.499
NC(cm)	29.40(2.73)	28.87(4.16)	33.47(5.03)	0.78	30.60(2.16)[a]	32.10(3.00)[a]	35.87(4.03)	<0.001*
WC(cm)	75.80(5.33)	76.61(6.60)	79.40(10.54)	0.73	84.81(7.44)[a]	86.23(5.38)[a]	89.00(5.26)	0.019*
WHtR	0.460(0.03)	0.475(0.04)	0.462(0.06)	0.5	0.538(0.04)	0.544(0.04)	0.559(0.03)	0.083
WHR	0.818(0.05)[a]	0.828(0.05)[a]	0.884(0.07)	0.02*	0.901(0.07)[a]	0.914(0.06)[a]	0.985(0.06)	<0.001*
R2D:4D	0.9537(0.02)	0.9634(0.02)	0.9585(0.03)	0.4	0.9720(0.02)[a]	0.9805(0.02)[a]	1.0013(0.03)	<0.001*
L2D4D	0.9518(0.02)	0.9640(0.02)	0.9585(0.03)	0.979	0.9711(0.02)[a]	0.9776(0.02)[a]	0.9905(0.02)	0.002*
Energy intake (kcal/day)	1630.00(362.43)	1707.58(295.00)	1730.00(442.34)	0.648	1750.00(413.95)[a]	1998.25(369.60)[a]	2483.08(413.29)	<0.001*
TSS(Minutes/week)	255.27(87.98)	374.85(140.05)[a]	411.00(161.28)[a]	0.001*	423.50(147.37)	517.54(133.35)[a]	592.31(131.37)[a]	0.041*
TPA**	2385.00(4177-405)	1300.0094124-40)	1674.50(2826-642)	0.002	511.50(4146-99)	582.00(3405-66)	557.00(1926-89.5)	0.918
VPA**	1485.00(1782-66)	660.0091782-0)	297.00(1386-33)	0.001	297.0091386-99)	264.0091782-0)	231.009792-33)	0.232
MPA**	720.00(1800-0)	320.0091760-0)	250.0091440-40)	0.005	90.00(960-0)	120.0091440-0)	100.00(360-0)	0.491
LPA**	320.00(1800-0)	80.0091440-0)	540.00(1800-0)	0.06	0(1800-0)	0(1680-0)	0(1800-0)	0.639

Data are expressed as mean (SD), ANOVA test (multiple comparability test Bonferroni) was used to compare means across genotype while Kruskal–Wallis test was used to test difference in physical activity.
BMI; Body mass index, NC; Neck circumference, WC; Waist circumference, WHR; Waist to hip ratio, WHtR; Waist to height ratio, R2D:4D, Ratio of right hand second to fourth digit, L2D:4D, Ratio of left hand second to fourth digit; TSS; Time spent sitting, TPA; Total physical activity, VPA; Vigorous physical activity, MPA; Moderate physical activity and LPA; Low physical activity.
**Data expressed by median (range), Kruskal–Wallis test (comparability test Dunns).

*Data expressed with same superscript in the same row are not statistically different at $p < 0.05$
